# Linkage- and ROH-based genomic relationship matrices for IBD based management of genetic diversity

**DOI:** 10.1186/s12711-026-01044-x

**Published:** 2026-03-30

**Authors:** Oda B. Wæge, Xijiang Yu, Peer Berg, Theo Meuwissen

**Affiliations:** Faculty of Life Sciences, Norwegian University of Life Sciences, 1432 Ås, Norway

## Abstract

**Background:**

The goal of managing genetic diversity is to maintain both the genomic uniqueness and adaptability of a population. This entails both maintaining heterozygosity and avoiding deleterious alleles from drifting to higher frequencies. Thus, genomic management strategies should not systematically change frequencies at neutral loci, as has been observed in current genomic management. We simulated 50 replicates of a population managed with optimal contribution selection (OCS), where inbreeding was controlled using different relationship matrices, where Van Raden method I (G_VR_), runs-of-homozygosity (G_ROH_), and identity by descent relationships (IBD) only used genomic information and a linkage analysis-based matrix (G_FGLA_) leveraged both genomic and pedigree information.

**Results:**

Across 50 simulated replicates, G_FGLA_ achieved the highest correlation with true IBD for both inbreeding and coancestry estimates and provided the most accurate rate of inbreeding (ΔF). G_FGLA_ also delivered the greatest genetic gain per unit of inbreeding and was the only scheme to remain below the inbreeding target while minimizing frequency change at neutral loci. G_VR_ using base population frequencies produced high genetic gain and controlled drift effectively but introduced systematic allele frequency changes towards the closest extreme frequency. Using current allele frequency resulted in high levels of inbreeding compared to the genetic gain achieved. G_ROH_ showed strong dependence on the minimum ROH length: short ROH improved ΔF estimation but increased allele frequency changes toward intermediate values, whereas long ROH underestimated inbreeding and reduced genetic gain.

**Conclusions:**

IBD-based matrices such as G_FGLA_ offer the most balanced approach for sustainable population management in OCS schemes, combining accurate relationship estimates, effective inbreeding control, and neutrality at neutral loci. In contrast, G_VR_ and G_ROH_ require calibration and cause systematic allele frequency changes.

## Background

After the implementation of genomic selection, generational of inbreeding in livestock populations have increased [1–4]. This increased rate of inbreeding, and the following loss of additive genetic variance can lead to a reduced selection response and make the population less adaptable for future breeding goals. In addition, an increased rate of inbreeding is associated with inbreeding depression, which is a phenomenon typically manifesting as increased rates of disease, reduced fertility and performance in production traits, and in severe cases extinction of the population [5]. Selection tends to move both selected alleles and those at linked loci toward fixation or loss [6]. Increasing population heterozygosity can counteract this process by maintaining allelic diversity [7, 8]. However, while maximizing expected heterozygosity promotes long-term genetic variation [9], it may also raise the frequency of deleterious alleles [10], thus causing a potential reduction in fitness over time. Additionally, when conserving small domestic populations with e.g. cultural value, or *ex-situ* species that will be reintroduced to the wild, maintaining genetic uniqueness, and by extension initial allele frequencies, is often one of the goals [11, 12]. Consequently, a sustainable management scheme needs to strike a balance between achieving genetic progress whilst maintaining initial frequencies at neutral loci.

In classic theory when inbreeding is calculated for neutral loci, which are not under selection, drift-based (F_drift_) and homozygosity-based (F_hom_) inbreeding are expected to be equal [13, 14]. A divergence between F_drift_ and F_hom_ under a given management strategy suggests that the management itself changes the allele frequencies and is therefore not neutral and does not conserve the population. A strategy to constrain the annual rate of inbreeding whilst maximising genetic progress in a population is optimum contribution selection (OCS) [15, 16]. Whilst pedigree based OCS is found to manage F_drift_ and F_hom_ equally [17, 18], OCS is dependent on accurate relationship matrices to function optimally [19], making it natural to use genomic relationships as pedigree information often is incomplete, susceptible to human error and does not account for the randomness of meiosis [20, 21]. However, Meuwissen et al. [18] found that OCS schemes using genomic relationships were not neutral with regard to the allele frequencies. The authors found that the genomic relationship matrices could be divided into three categories where VanRaden I (G_VR_) and II managed drift, while the ROH-matrix and the G_VR_ using 0.5 as reference frequencies managed homozygosity, resulting in F_drift_ no longer being equal to F_hom_ for these OCS schemes. The authors saw however promising results from a linkage based genomic relationship matrix (G_FGLA_) that used both genomic and pedigree data. The scheme implementing this relationship matrix managed both F_drift_ and F_hom_. Therefore, due to the accuracy issues with pedigrees, and the lack of neutrality in the current genomic matrices there is still a need to find a genomic relationship matrix that holds the same properties as the numerator relationship matrix.

The pedigree based inbreeding coefficient estimates the genome-wide average Identity-by-descent (IBD), or the probability that the two alleles at any given neutral locus stem from the same common ancestral allele [22]. Genomically IBD is expected to appear as stretches of homozygous loci called runs-of-homozygosity (ROH) [23]. Many methods have been developed to find ROH with some common detection algorithms being rule based, hidden Markov models, hashing and extensions, and different adaptions of the positional burrows-Wheeler transformation [24]. A common issue with these detection methods is finding the right compromise between detection of all short IBD segments, without also detecting Identity-by-state (IBS) homozygous haplotypes [25]. Capturing these shorter segments can however be important as the length of IBD segments can be used to estimate historic inbreeding events [23]. Chromosomal segments are over time broken up by recombination during meiosis. This implies that from a common ancestor $t$ generations back in time the resulting length of segments will be exponentially distributed with a mean length of $1/(2t)$ Morgan [26]. Thus, choosing length of segments to include becomes a balancing act between accuracy and historical depth.

The goal of this study is to compare the inbreeding management in selection schemes using relationship matrices based on either linkage analysis (G_FGLA_); ROH (G_ROH_) or genomic covariance (G_VR_) in computer simulations of OCS schemes. In addition, it is investigated whether the selection results in the rate of genetic drift exceeds that of the rate of homozygosity, which implies an increased loss of rare alleles relative to the rate of inbreeding, or *vice-versa*. Moreover, the effect of ROH length on genomic management will be examined.

## Methods

### Simulation

A historic population was generated using Msprime 1.2.0 [27]. We simulated a population with a constant effective population size (${N}_{e}$) of 1000, and a synthetic species with 30 equal length autosomes totalling to a 3 Gbp long genome. The coalescent simulation proceeded backwards in time without an imposed time limit, stopping once complete coalescence was reached. This was when all lineages merged into a single most recent common ancestor at every genomic position. This ancestral tree sequence was subsequently used as the historic population for the forward-in-time stochastic simulations. All chromosomes were of equal length and had a mutation and recombination rate of $2.2\times {10}^{-9}$ and $1\times {10}^{-8}$ per base pair per meiosis, respectively.

For simulating our selection schemes we used the forward in time stochastic simulation software xyBnG [28]. From the historic population three groups of biallelic loci with a MAF over 1% were randomly sampled. These groups were 50 000 SNPs for the SNP-chip, 10 000 reference loci and 10 000 QTLs (quantitative trait loci). The reference loci are neutral in the sense that they are not used for the genetic estimation nor management. These neutral markers were used to monitor the neutral diversity and drift in the population during selection. The QTLs contributed to a trait with a heritability of 0.25. QTL effects were initially sampled from a normal distribution and then scaled so that the resulting true breeding values (TBV) across individuals also followed a standard normal distribution. While this section of simulation was subject to a recombination rate of $1\times {10}^{-8}$ per base pair per meiosis, no new mutations were simulated.

To generate a base population with traceable IBD xyBnG simulated 10 generations of random mating with a sample of individuals from our historic population. Here for each replicate 100 males and 100 females were randomly selected to produce 400 offspring to generate the base population. For every SNP, neutral loci and QTL have, in addition to the normal alleles, also a set of founder alleles. These founder alleles are uniquely coded for each of the founder animals sampled from the historic population and are inherited by their offspring to calculate true IBD.

From this base population we simulate selection for 10 generations using OCS [16] for inbreeding management, with a target rate of inbreeding of 0.5% per generation. Since the inbreeding was controlled using different genetic relationship matrices, the average expected inbreeding in the base population (the generation in which OCS starts) was also calculated using the relationship matrix used by the OCS method.

In the present study, our primary focus is on how different methods of inbreeding management affect selection outcomes. To isolate this effect, EBVs in all selection schemes, except one (G_IBD-FULL_), were estimated using GBLUP [29] with VanRaden I genomic relationship matrix (G_VR_) [30] where the genotypes being centred using base population allele frequencies. While there are other molecular matrices (e.g. VanRaden method II and III), we only apply one here to both represent the molecular methods, and the current industry standard.

The G_IBD-FULL_ scheme differs from the others in that both EBV estimation and inbreeding management relied on the true IBD relationship matrix (G_IBD_), rather than on GBLUP. Its selection accuracy was slightly lower than that of the scheme that estimated EBVs using GBLUP while constraining inbreeding with true IBD. This suggests that linkage-analysis-based EBVs are less accurate, likely because the pre–base population relationships captured in the G_VR_ improved accuracy in the GBLUP-based schemes.

The diagonal and off-diagonal elements of the G_IBD_ matrix were computed as the proportion of SNP loci homozygous for founder alleles across all possible gamete combinations, divided by twice the number of loci. For the off-diagonal elements all gametic combinations between two individuals represents all potential parental combinations, similarly to how a gametic based relationship matrix is constructed [31]. This provides a true relationship matrix for further benchmarking.

To simplify the naming of the schemes, we therefore label them according to the matrix used to constrain inbreeding. Inbreeding was constrained using one of several relationship matrices: the G_VR_ based on current allele frequencies (G_VR_CF_) or base-population frequencies (G_VR_BF_), the G_IBD_ matrix, the G_FGLA_ matrix, or ROH-based matrices with different minimum ROH length thresholds (G_ROHX_, where $X$ denotes the minimum ROH length in cM).

To produce the designated number of offspring, the offspring for the next generation were generated by random sampling of parents proportional to the optimal contributions, and within parents’ gametes were randomly sampled with random assortment of chromosomes and recombination following a Poisson process (Haldane 1919).

### Runs-of-Homozygosity relationship matrix (G_ROH_)

The Runs-of-homozygosity relationship matrix (G_ROH_) was constructed using the framework from de Cara et al. [32]. First the ROH were detected using the software *phasedIBD* from Freyman et al. [33]. As there are no genotyping or phasing errors in simulated data, we detected the ROH using the *TPBWTAnalysis* function with template option “[[1]]” that collapses the model down to a PBWT (Positional Burrows Wheeler Transformation) model, that does not allow for errors. Each ROH needed a minimum of 20 sequentially homozygous SNPs, and was a minimum length of either 1, 5, 8 or 10 cM.

The resulting segment file from *phasedIBD* includes both IBD segments within one individual and segments between the haplotypes of two individuals. To find shared IBD segments between two individuals *phasedIBD* detects segments for all (2 × 2) haplotype combinations. Within a haplotype combination *phasedIBD* at times produces overlapping segments, causing an overestimation of IBD. We therefore scanned through the segments and combined overlapping segments.

Using the formulas presented in de Cara et al. [32] the diagonal elements of the G_ROH_ for individual *a* was calculated as

1 $${G}_{ROH}(a,a)=1+ \frac{\sum {L}_{se{g}_{k}}({a}_{1},{a}_{2} )}{L},$$

where ${{L}_{seg}}_{k}$ is the length of segment $k$ on individual $a$*’*s gametes. $L$ is the total observable length of the genome. The estimated relationship between individual $a$ and $b$ was calculated as

2 $$ \begin{gathered} G_{{ROH}} \left( {a,b} \right) = \hfill \\ \frac{{\sum {L_{{seg\,k}} } \left( {a_{1} ,b_{1} } \right) + \sum {L_{{seg\,k}} } \left( {a_{1} ,b_{2} } \right) + \sum {L_{{seg\,k}} } \left( {a_{2} ,b_{1} } \right) + \sum {L_{{seg\,k}} } \left( {a_{2} ,b_{2} } \right)}}{{2L}}, \hfill \\ \end{gathered} $$

where ${L}_{se{g}_{k}}\left(a, b\right)$ is the length of segment k on each of the haplotype pairs $\left({a}_{1},{b}_{1}\right),\left({a}_{1},{b}_{2}\right),\left({a}_{2},{b}_{1}\right)$ and $\left({a}_{2},{b}_{2}\right)$ from individuals $a$ and $b.$

### Linkage based relationship matrix G_FGLA_

The idea of using linkage analysis was first presented by Fernando and Grossman [34], which was later adapted and used in the simulation study by Meuwissen et al. [18]. The version of G_FGLA_ in the present study is a more efficient version of that methodology and is described in detail in Meuwissen et al. [35], but is available as a GitHub repository [36]. In brief, the method uses the 50 K SNP data to infer segregation indicators, which distinguish paternal from maternal inheritances of alleles at the SNP loci. Unknown segregation indicators are imputed using known indicators on the same chromosome, or if the latter are not available, the indicators are randomly sampled. Sampling errors tend to average out here due to the large number of loci considered (50 K). Next, unique founder alleles are allocated to the base population animals, and these alleles are gene-dropped through the pedigree using the known and sampled segregation indicators. Lastly, G_FGLA_ relationships are calculated based on the identities of these unique founder alleles and averaged over all loci.

### Measures of inbreeding

To examine the effect of the different relationship matrixes we stored information about number of parents selected, mean true and estimated breeding values, and number of QTL alleles lost and fixed. We also calculated the true inbreeding, estimated inbreeding based on drift (${F}_{drift}$) and homozygosity ${F}_{hom}$. ${F}_{drift}$ was calculated per locus $i$ as

3 $${F}_{drif{t}_{i}}=\frac{2{\left({q}_{t,i}-{q}_{0,i}\right)}^{2}}{He{t}_{0,i}},$$

where$He{t}_{0,i}= 2{q}_{0,i} (1-{q}_{0,i} )$, and represents the heterozygosity at locus $i$ in generation 0. ${q}_{t,i}$ and ${q}_{0i}$ are the allele frequency at locus $i$ in generation $t$ and 0 respectively. The F_drift_ values where averaged across loci. ${F}_{hom}$ was then calculated per locus $i$ as

4 $${F}_{{\mathrm{hom}}_{i} }= \frac{He{t}_{0,i}-He{t}_{t,i}}{He{t}_{0,i}}.$$

And subsequently averaged across loci.

### Variance estimates

The genic variance (${\sigma }_{a}^{2}$) was measured as

5 $${\sigma }_{a}^{2}=2{\sum }_{i}{p}_{i}{q}_{i}{a}_{i}^{2},$$

where ${a}_{i}$ is the known additive effect of locus $i$ [37]. The genetic variance (${\sigma }_{u}^{2}$) was calculated as the variance of true breeding values (TBV) across individuals.

6 $${\sigma }_{u}^{2}=var(TBV),$$

where the true breeding value of individual $j$ is $TB{V}_{j}={\sum }{x}_{ij}{a}_{i}$ with ${x}_{ij}$ being the number of reference alleles (0, 1, or 2) at QTL $i$ for individual $j$.

### Comparing coancestry and inbreeding coefficient estimates

To compare the quality of inbreeding and coancestry estimates the genotypes from the 50 replicates of the G_IBD-FULL_ scheme were extracted. The genomic relationships were computed for the G_IBD_, G_VR_CF_, G_VR_BF_, G_FGLA_ and the different minimum length G_ROH_. To evaluate how well the matrices ranked the relationships in contrast to the true values from the G_IBD_ we regressed the estimated relationships on the true IBD values. The inbreeding coefficients and the relationship values were regressed separately. To evaluate the absolute agreement between G_IBD_ the estimates we also calculated Lin’s concordance correlation coefficient [38].

### Comparing the rate of inbreeding estimates

We used these same coancestry calculations, i.e. half the relationships, to also compare how the different relationship matrices estimate the rate of increase of coancestries ($\Delta f$). While it is more common to calculate rate of inbreeding per year, the present study calculates rate of inbreeding per generation as the software produces per generation genotypes. The rate of coancestries was estimated as the slope of $-\mathrm{log}\left(1-{f}_{t}\right)$ regressed on generation t. Where ${f}_{t}$ is the average level of coancestry at generation t. This method was used since, as Hinrichs [39] and Meuwissen [18] explains, when ${f}_{t}=1-{\left(1-\Delta f\right)}^{t}$ and $\Delta f$ is constant, $-\mathrm{log}\left(1-{f}_{t}\right)$ will change linearly over time with a slope of$-\mathrm{log}(1-\Delta f )\approx\Delta f$.

## Results

### Comparison of genetic relationship matrices

Tables 1 and 2 show the relationship between estimated and true inbreeding (diagonals) and coancestry (off-diagonals) across all generations and replicates. Differences among matrices were more pronounced for the diagonal than for the off-diagonal elements and is mainly explained by the different volume of observations. For the diagonals, G_FGLA_ showed greater precision and ranking ability resulting in an almost perfect concordance correlation coefficient (CCC). While G_VR_BF_ had a slope closer to one the greater variance and lower correlation cause G_VR_BF_ to be outperformed by G_ROH05_, G_ROH08_ and G_ROH10_ for CCC. For the off-diagonals, slopes were nearly identical across methods, but G_FGLA_ still provided higher CCC. Among the ROH matrices, higher thresholds (G_ROH05_, G_ROH08,_ G_ROH10_) approached the correct scale but still lagged behind G_FGLA_ in overall CCC agreement. For both measures G_VR_CF_ had some of the lowest correlation and CCC scores, showing the matrices inability to measure the right magnitude of inbreeding, and correctly rank the animals.

**Table 1:** Regression estimates of inbreeding estimates regressed on true inbreeding (G_IBD_)

Relationship matrix	intercept	Slope	SE	Correlation	CCC
G_IBD_	0.0000	1.0000	0.00000	1.0000	1.0000
G_VR_BF_	-0.0070	1.0064	0.00364	0.8904	0.8838
G_VR_CF_	0.3596	0.9057	0.00328	0.8383	0.2739
G_FGLA_	-0.0174	1.0171	0.00069	0.9955	0.9949
G_ROH01_	0.2270	0.8948	0.00195	0.9558	0.1327
G_ROH05_	0.0063	1.0068	0.00147	0.9794	0.9067
G_ROH08_	-0.0093	1.0032	0.00137	0.9820	0.9670
G_ROH10_	-0.0016	0.9885	0.00147	0.9787	0.9031

Values in the table represents the diagonal elements of the relationship matrix of the final generation. Intercept, Slope and Standard error is estimated by a linear model. Correlation and Lin’s concordance correlation coefficient (CCC) is calculated from the data

**Table 2:** Regression estimates of relationship estimates regressed on true relationships (G_IBD_)

Relationship matrix	Intercept	Slope	SE	Correlation	CCC
G_IBD_	0.0000	1.0000	0.00000	1.0000	1.0000
G_VR_BF_	-0.0002	1.0000	0.00007	0.9835	0.9833
G_VR_CF_	0.1466	0.9057	0.00008	0.9688	0.2547
G_FGLA_	0.0003	0.9998	0.00002	0.9988	0.9988
G_ROH01_	0.2447	0.8861	0.00007	0.9787	0.1087
G_ROH05_	0.0275	0.9978	0.00004	0.9922	0.9000
G_ROH08_	-0.0130	1.0080	0.00004	0.9937	0.9749
G_ROH10_	-0.0283	1.0054	0.00004	0.9926	0.8992

Values in table represents the off-diagonal elements of the relationship matrix of the final generation. Intercept, Slope and Standard error (SE) is estimated by a linear model. Correlation and Lin’s concordance correlation coefficient (CCC) is calculated from the data

### Estimating the rate of inbreeding

To evaluate how well the different relationship matrices estimated the rate of inbreeding we calculated $\Delta F$ with the individuals from the 50 G_IBD-FULL_ replicates. Table 3 shows that the methods that use molecular information and smaller segments like G_VR_BF_, G_FGLA_ and G_ROH01_ approximate true $\Delta F$ (G_IBD_) the closest, with G_FGLA_ having a nearly perfect estimate. When comparing just the G_ROH_ there is a clear tendency that the higher the threshold of ROH length the greater the underestimation of $\Delta F$ is, which was also found by Wæge et al. [40]. Allele frequency also had a large effect on the rate of inbreeding estimates from G_VR_, with the usage of base population frequencies resulting in almost correct estimates, while the current allele frequencies resulted in underestimation. This discrepancy may be a result of the matrix with the current frequency not being able to capture the accumulation of drift over time.

**Table 3:** Average ${\boldsymbol{\Delta}}\mathbf{F}$ (Rate of inbreeding) and standard error (SE) estimated using different relationship matrices for the same 50 populations for 20 generations

Relationship matrix	${\boldsymbol{\Delta}}$ F	SE
G_IBD_	0.00375	0.00015
G_VR_BF_	0.00379	0.00015
G_VR_CF_	0.00058	0.00023
G_FGLA_	0.00375	0.00015
G_ROH01_	0.00374	0.00016
G_ROH05_	0.00342	0.00014
G_ROH08_	0.00300	0.00011
G_ROH10_	0.00272	0.00009

### Number of selected parents and genetic response

**Fig. 1:**
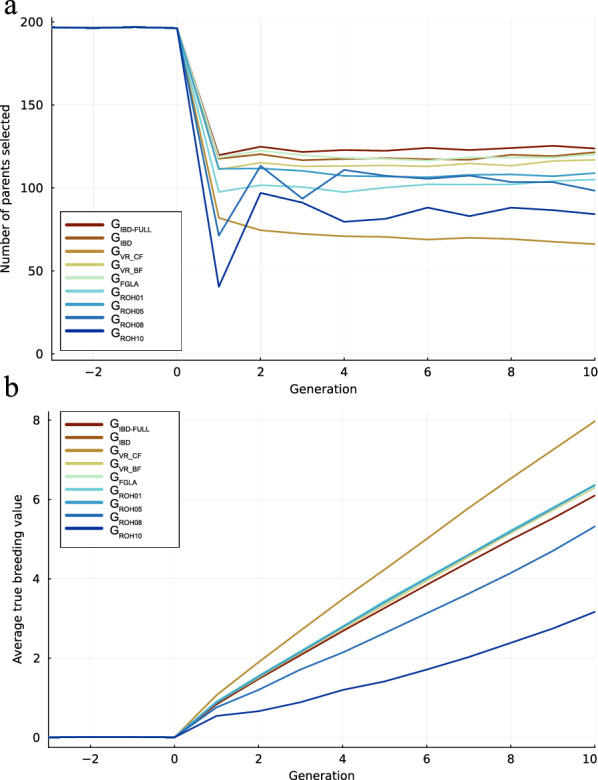
Number of selected parents and true breeding value. **a** the number of animals selected for each generation. The colour indicates the relationship matrix used. **b** The average true breeding value for all generations. The colour indicates the OCS scheme

Figure 1a illustrates the number of parents selected for the next generation. The figure shows that G_IBD-FULL_ selects the largest number of parents, closely followed by G_IBD_, G_FGLA_ and G_VR_BF_. Overall, for the G_ROH_ scenarios G_ROH10_ selects fewest parents followed by G_ROH08_, G_ROH01_ and finally G_ROH05_. The fewest parents were selected by G_VR_CF_. For genetic response (Fig. 1b) G_VR_CF_ produced the highest mean TBV (7.97) in generation 10, followed by G_ROH01_ (6.37), G_ROH05_ (6.36), G_IBD_ (6.33), G_FGLA_ (6.32), G_VR_BF_ (6.30), and G_IBD-FULL_ (6.10). G_ROH08_ and G_ROH10_ had the two lowest mean TBV (5.32 and 3.17) and is not considered further in the Results section.

#### Inbreeding

**Fig. 2:**
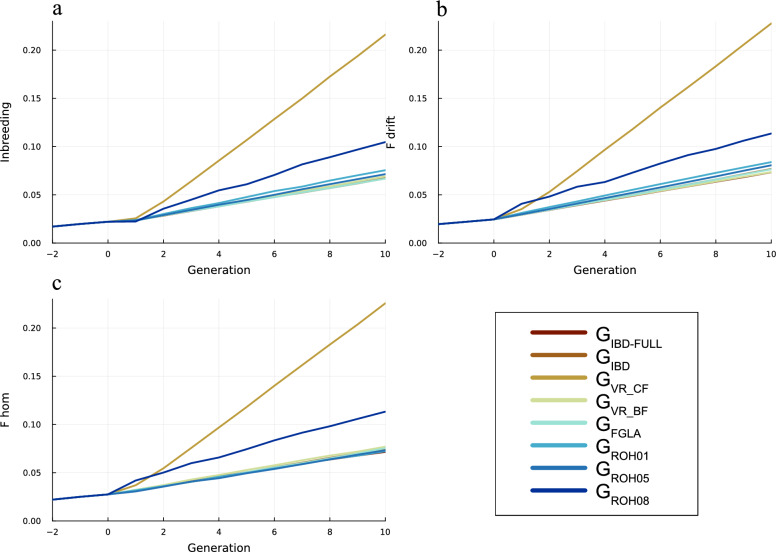
Average IBD, drift and homozygosity inbreeding. Average inbreeding for each generation. **a** is the true inbreeding, **b** is drift-based inbreeding at neutral loci, and **c** is homozygosity-based inbreeding at neutral loci. The colours indicate which relationship matrix was used

Figure 2 depicts the true, drift and homozygosity-based inbreeding. In Fig. 2a of true inbreeding (${F}_{IBD}$), G_FGLA_ has the lowest inbreeding by generation closely followed by G_IBD_ and G_IBD-FULL_. G_ROH_ schemes G_ROH05_ had the lowest IBD inbreeding in the final generation, with G_ROH01_, and G_ROH08_ generating increasingly more inbreeding. For ${F}_{drift}$ (Fig. 2b) G_VR_BF_ achieves the lower ${F}_{drift}$ after G_IBD-FULL_. There is however a reranking of schemes looking at ${F}_{hom}$ (Fig. 2c) where G_IBD-FULL_, G_ROH01_ and G_ROH05_ reduce inbreeding the most followed by G_IBD_, G_VR_BF_, G_FGLA_ and G_ROH08_ in that order. It seems that the G_ROH01_ and G_ROH05_ schemes reduce F_hom_ substantially, which implies striving towards intermediate allele frequencies (0.5), but this is at the expense of increased genetic drift (allele frequency changes). G_VR_CF_ had the highest increase in inbreeding for all measures.

### Neutral management

**Fig. 3:**
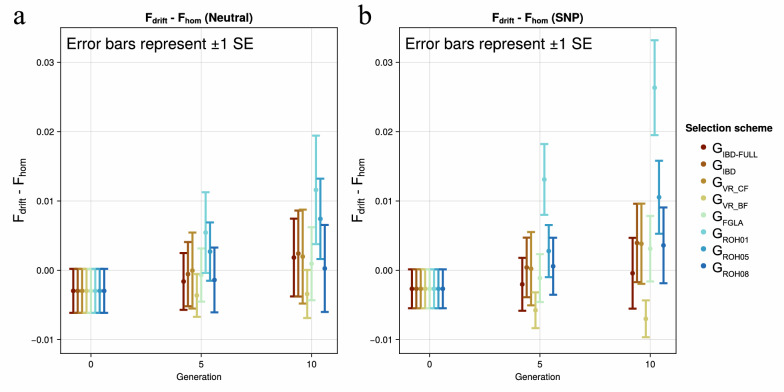
Difference between F_HOM_ and F_drift_. The difference of F_drift_—F_hom_ over time. The Selection scheme is represented by the line colour. Figure **a** shows the neutral markers and **b** the SNPs

Here, we investigated the neutrality of management schemes in the sense that the inbreeding management does not systematically affect allele frequencies. Meuwissen et al. [18] show that the difference (${F}_{drift}-{F}_{hom}$) is proportional to the covariance between allele frequency change and initial frequency. For instance, F_hom_ < F_drift_, as in case of the G_ROH01_ and G_ROH05_ schemes, indicates a negative covariance between initial frequencies and frequency changes and thus rare alleles are changing towards intermediate frequencies, which poses a risk in the presence of rare deleterious mutations. Figure 3 a and b show the difference between F_drift_ and F_hom_ for neutral loci and SNPs. For the neutral loci there is only a significant difference between G_VR_BF_ and G_ROH01_ and G_ROH05_ in generation 10. For the SNPs there is a greater difference between the schemes with especially G_ROH01_ being significantly different from all the other schemes. This indicates that the method is moving the allele frequencies towards 0.5. As expected, the schemes that are based on true IBD or stronger proxies for IBD (e.g. G_FGLA_ and G_ROH08_) are the closest to achieving neutral management by not being significantly different from zero. Unexpectedly G_VR_CF_ was not significantly different from 0 indicating that when the reference allele frequency is updated each generation, large frequency changes are not punished, thus not having a stronger reduction of drift-based inbreeding.

### Loss of variation

**Fig. 4:**
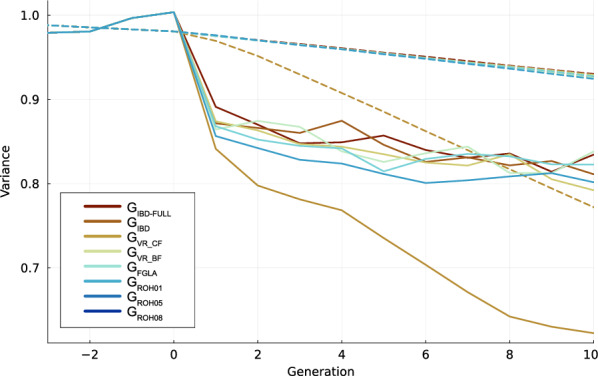
Change in genetic diversity. Variance on breeding values (solid lines) and the genic variance (dotted lines) for the different selection schemes indicated with colour

Figure 4 shows the decline in genic variance and variance of true breeding values. For genic variance we see that the G_ROH08_ and G_VR_CF_ is losing significantly more genic diversity than the other methods. For the upper cluster of schemes there is no significant difference. For variance of true breeding values, which would also represent genetic diversity there is a greater difference between methods but G_ROH08_ and G_VR_CF_ is still the only scheme that significantly deviates from the other methods.

### Genetic gain versus inbreeding

**Fig. 5:**
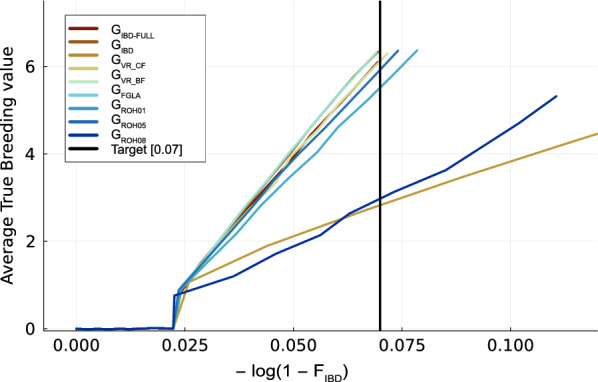
Genetic gain against inbreeding. Average True breeding value plotted against accumulated inbreeding. The vertical black line indicates target level of inbreeding when selecting with a 0.05 constraint

Figure 5 plots genetic gain versus the true inbreeding. Here G_IBD-FULL_, G_IBD_ and G_FGLA_ are able to maintain true inbreeding below our final target at generation 10 of 0.07. The target was calculated as the already accumulated inbreeding (F_IBD_) after 10 generations of random breeding (0.02) plus the inbreeding constraint times the number of generations of selection. G_FGLA_ and G_IBD_ had the most genetic gain per unit inbreeding with a slope of 98.83 and 98.61 respectively, followed by G_VR_BF_ (95.95), G_IBD-FULL_ (94.72) and then G_ROH_ schemes. For the G_ROH_ schemes we see the same tendency as previous results with 5 cM being the most akin to G_IBD-FULL_ and G_VR_BF_, while G_ROH01_ does slightly worse and G_ROH08_ severely underperforms. Similarly to G_ROH05_, G_VR_CF_ had a slope of 35.63. When true breeding values are plotted against F_drift_ based on neutral loci we see the same ranking of methods as in Fig. 4 (result not shown). For F_drift_, however, none of the methods stay below the inbreeding target. For F_hom_ (inbreeding target after 10 generations given the initial F_Hom_ inbreeding = 0.75) the ranking changes with only G_IBD-FULL_ and G_ROH01_ achieving the inbreeding constraint.

## Discussion

In this study, we explored how different genomic relationship matrices affect population management. We simulated 50 replicates of a population managed with optimal contribution selection (OCS), where inbreeding was controlled using different genomic relationship matrices based on Van Raden method I, ROH, linkage analysis and true IBD relationships. Previous research has shown that the traditional pedigree-based A-matrix can achieve higher genetic gain [17] and maintain allele frequencies more neutrally than genomic matrices, but it fails to adequately control the rate of inbreeding [3, 17, 18]. Therefore, our focus in the present study was on comparing genomic matrices and evaluating their ability to maintain equivalence between drift and homozygosity-based inbreeding at loci not under selection.

Accurate estimation of genetic relationships is essential for effective population management. While our results indicate that all presented methods have the potential to correctly rank individuals based on both inbreeding and coancestry (all correlations > 0.97). The CCC results also indicates that all methods beside G_ROH01_ and G_VR_CF_ are also able to estimate the correct magnitude of relatedness. While previous studies have demonstrated that ROH-based measures are effective for estimating inbreeding [2, 30, 31], in the present simulation study we found G_ROH_ to be outperformed by G_FGLA_ for both inbreeding and coancestry estimates with both higher correlations and CCC values. We do however see in the present study that the lengths of ROH considered influences the results. This was also argued by Caballero et al. [41] where they found that setting the lower threshold to what would mainly cover inbreeding since the creation of the base population resulted in the best estimates of F_IBD_. We found G_ROH_ to also be outperformed by G_VR_BF_, but in comparison to G_FGLA_, G_VR_BF_ was less precise (Table 1 and 2). Here we assumed that F_IBD_ reflects true IBD, based on a well-defined base population, but in real-life populations, with overlapping generations, missing parentages, etcetera, the definition of the base population may be complicated, and also other natural forces may affect the Mendelian segregation of chromosomal segments, which implies that the differences between the methods may be less clear cut than in our simulation study.

For rate of inbreeding, G_FGLA_ provided the most accurate estimates (Table 3). A common property for all the top-performing methods (e.g. G_FGLA_, G_VR_BF_, G_ROH01_) was the use of more detailed molecular level information, i.e. information on individual SNPs or small instead of large haplotypes. This may be because homozygosity of individual SNPs and small haplotypes captures better the cumulative nature of inbreeding as recombination breaks up longer ROH. The cumulative nature of inbreeding is also made clear by the difference between G_VR_BF_ and G_VR_CF_. When the frequency is updated every generation the change in allele frequencies is not being correctly weighted, meaning that the matrix produces an underestimation of inbreeding in the population, as was also seen with the CCC scores.

When applied in a selection scheme, how these matrices perceive distance between individuals affects both the number of animals selected and the genetic similarity among those selected. We see for example that both G_ROH08_, G_ROH10_ and G_VR_CF_ which underestimates the relationships also are the methods that select the fewest parents (Fig. 1). In contrast the methods that have the highest CCC scores when compared to true relationships are the ones that also select the most parents each generation. What is however notable is that G_ROH01_, which is the method with the largest overestimates of relatedness, also selects relatively few animals. However, this discrepancy may be explained by G_ROH01_ being better at selecting genetically distant animals as the method is able to maintain a high variation of variance in the breeding values (Fig. 4). This plot of breeding value and genic variance shows the steady decline genic variation, while the breeding value variance shows the steeper decline related to the Bulmer effect. While most of the presented methods are quite similar, G_VR_CF_ results in much less variance over time, showing again the consequence of underestimating relationships.

The ability of G_FGLA_ to correctly rank the coancestries of the individuals in our populations resulted in it achieving the most genetic progress per unit IBD inbreeding. G_FGLA_ was also the only scheme that was able to stay below our inbreeding target (Fig. 5). While G_FGLA_ had the lowest level of IBD inbreeding, G_VR_BF_ restricted drift the most, and G_ROH01_ had the best control of homozygosity, which indicate that G_VR_BF_ and G_ROH_ were not able to manage the population neutrally with regard to independence of initial allele frequency.

A secondary goal of this study was to compare the ability of the different genomic matrices to avoid drift at neutral loci in the genome. Both this study and a previous study by Meuwissen et al. [18] found that most genomic relationship matrices are not neutral when used for inbreeding management. The G_VR_BF_ favours allele frequency changes of the minor allele towards the nearest extreme, although this effect was less extreme here than in Meuwissen et al. [18] due to the large, realistic genome size used in the current study. It is however notable that G_VR_CF_ did not produce a significant difference between drift and homozygosity-based inbreeding, which may be a result of the matrix not adding additional weight to the alleles drifting away from the initial frequency. The G_ROH_ matrices moved the frequency of neutral loci towards intermediate values, but the effect was less severe as the ROH included became longer. G_FGLA_ was however able to maintain neutrality, indicating it achieved IBD based management.

All schemes were evaluated under idealized conditions. ROH-based estimation of relationships relies on knowing the phase of haplotypes. While we used true phases of the haplotypes, for a real population we would expect different levels of switch errors depending on the phasing method and the training population size for the phasing [32], which consequently would reduce the accuracy of relationship estimates. Similarly, G_VR_BF_ had access to the true initial allele frequencies which has been shown to increase the accuracy of Van Raden I [33]. For real populations these must be estimated, thus reducing the quality of the estimates. As shown in Meuwissen et al. [35] the quality of the relationship estimates from G_FGLA_ depend on the number of genotyped animals in the population, especially regarding numbers of genotyped generations back in time. In our simulations, all animals born after the base-population were genotyped, maximizing G_FGLA_’s potential.

Another parameter that can affect the quality and informativeness of ROH is the marker density. While the historical depth of the simulated population would result in longer ROH as mentioned above, using a denser SNP chip would allow for both detecting shorter ROH, but also having more SNPs per ROH thus achieving a higher accuracy [42]. While including shorter ROH may have improved the rate of inbreeding estimates, multiple studies have found them to more often be representative of IBS rather than IBD [25, 40], further exemplifying the need for case-by-case calibration when applying ROH based relationships.

Classic inbreeding theory is based on loci that are not affected by selection, in other words they are neutral and selection free [19]. In this present study we defined neutral loci as loci that were not visible SNPs and not used for OCS, and we used these neutral loci to examine the change in inbreeding. However, at 50 k SNPs, as in the current study, it is expected that all loci are at least in partial linkage disequilibrium (LD) with the SNPs under selection [43]. We can see from Fig. 3 that the neutral loci and the SNPs behave similarly due to their LD, but allele frequency changes and inbreeding is slightly less at the neutral loci.

A reason for why G_FGLA_ outperforms the other methods may be that the matrix is only able to evaluate the inbreeding produced after the creation of the historic population, due to the segregation loci being a product of both pedigree and genomic information. The importance of filtering away pre-base population inbreeding is made especially clear when we look at genetic gain (Fig. 5). Here we see that using base population allele frequencies are much more efficient at managing inbreeding compared to using current allele frequencies for G_VR_. This is also true for G_ROH_, where using a minimum length of 5 cM, which is the expected mean length of ROH for 10 generations since common ancestor, resulted in the most genetic gain per unit inbreeding. In addition, for genomic regions or individuals lacking segregation loci G_FGLA_ resorts to using only pedigree information. As shown in a previous study pedigree-based OCS is both highly efficient in regard to genetic gain, but it is also inherently IBD-based [18]. This means that G_FGLA_ will always be IBD-based, regardless of number of genotyped generations. Given that both our measures of correct estimation and neutrality are IBD-based, G_FGLA_ naturally exhibit strong performance in the present study.

Overall, our findings highlight the differences among genomic relationship matrices in their ability to manage inbreeding, genetic drift, and allele frequency changes under OCS. G_FGLA_ consistently provided the most accurate estimates of IBD, enabling effective control of inbreeding while minimizing frequency change at neutral loci. In contrast, G_VR_ and G_ROH_ caused systematic changes to allele frequencies, despite estimating relationships reasonably well. These results emphasize the importance of selecting relationship matrices that capture true IBD information to balance genetic progress and maintenance of long-term genetic plasticity.

## Conclusions

Our results demonstrate that genomic relationship matrices differ in their ability to manage inbreeding and genetic diversity under OCS. G_FGLA_ provided the most accurate estimates of relationships and inbreeding, achieved the highest genetic gain per unit of inbreeding, and was the only method to achieve the desired rate of inbreeding at neutral loci. In contrast, G_VR_, although effective in controlling drift and achieving high genetic gain, introduced systematic allele frequency changes. G_ROH_ showed strong dependence on the chosen ROH length threshold, with short ROH favouring homozygosity control at the expense of drift and long ROH underestimating inbreeding. These findings indicate that G_FGLA_ may offer an accurate IBD-based measure of inbreeding, and thus improved OCS based sustainable population management.

## Data Availability

The data utilized in this study was generated through simulations and does not include any proprietary or confidential information. The scripts used for these simulations and data analysis are publicly available and can be accessed in the GitHub repository (https://github.com/odawage/IBD_OCS/). For further inquiries or additional information, please refer to the corresponding author.
